# End of life care for long-term neurological conditions: A meta-ethnographic review of the experiences of informal carers

**DOI:** 10.1177/0269216320974262

**Published:** 2020-11-25

**Authors:** Michael Toze, Mo Ray, Thomas George, Kelly Sisson, David Nelson

**Affiliations:** 1 Lincoln Medical School, University of Lincoln, Lincoln, UK; 2 School of Health and Social Care, University of Lincoln, Lincoln, UK

**Keywords:** Neurological, palliative, end-of-life, carers, family

## Abstract

**Background:**

Family and friends are key providers of care for people living with a long-term neurological condition. Neurological conditions are a significant global contributor to disability and premature death. However, previous research suggests carers often struggle to access appropriate support at end of life.

**Aims:**

This review sought to synthesise qualitative studies discussing end-of-life and palliative issues for informal carers supporting people living with neurological conditions.

**Design:**

This was a meta-ethnographic synthesis of 38 qualitative studies discussing end-of-life and palliative issues for informal carers supporting people living with long-term neurological conditions.

**Data Sources:**

Qualitative articles published after January 2010 in English, addressing carers of people with long-term neurological conditions with regard to palliative care, end of life and/or bereavement. Papers were excluded if it was not possible to separately assess the views of carers. Quality appraisal was not undertaken, but consideration was given to research context.

**Results:**

Across the papers, five key themes were identified: the future (un)certainties in the progression of life-limiting neurological conditions; an information paradox of not receiving the right information at the right time; access to support; carers’ roles in decision making around end of life; and maintaining continuity while facing change and disruption in day-to-day living.

**Conclusions:**

Given the broad agreement on the challenges faced by carers of people living with long-term neurological conditions, future research should consider opportunities to improve information and support for this group, and the development and evaluation of practical models of service delivery.

**What is known about this topic?**

- Neurological conditions are a significant global contributor to disability and premature death
- Carers face particular challenges regarding the uncertainty of conditions and difficulty in accessing support

**What this review adds?**

- The review identifies key themes in the existing literature relating to future (un)certainties; information paradox; access to support; decision making; and continuity, change and disruption.

**Implications for policy and practice?**

- There is a need for better communication about the role of palliative care.
- Given the relatively clear narrative regarding existing challenges and barriers, future research should address practical approaches to improving services, including models for service delivery.

## Introduction

Neurological conditions are the leading global contributor to Disability Adjusted Life Years (DALY), a measure of the impact of disease on life expectancy and years spent living with disability.^1^ In the UK, neurological conditions account for 20% of acute hospital admissions and are the third most common reason for people to access primary care.^2^

Research suggests that family carers of people with life-limiting neurological conditions face a number of distinct challenges linked to the long-term nature of the conditions, the physical, cognitive and behavioural challenges, and the consequent care requirements.^3,4^ Caring for a person with neurological conditions may be made additionally complex by factors such as other co-existing conditions, broader social inequalities, individual illness experience, and the degree of access to formal and informal support.^3,5–7^ Carers of people living with long-term neurological conditions are likely to experience caregiving as intensive or ‘relentless’ and may be critical of services where they feel that the emotional implications of caring for someone who will die of their condition are not properly recognised or supported.^8–10^ Specific challenges have been associated with end of life care for people with neurological conditions and their carers and families, including living with complex conditions and uncertain disease trajectories, the lack of a distinct dying phase for some conditions and poor access to palliative care services.^11^

Given that long-term neurological conditions are characterised by change and deterioration, people living with such conditions and their families engage with multiple health and care service systems. Responsive, easy to navigate services, combined with clear information and advice, are important markers of quality. However, health and social care practitioners may find it difficult to practice confidently for a variety of reasons, including the challenges of developing the skill and knowledge base required to respond to complex conditions that may only be seen infrequently.^12–14^ There are a number of pre-existing qualitative studies addressing challenges faced by informal carers of people with neurological conditions. Many of these studies address end-of-life care, but are usually disease- and/or setting-specific and often consider end-of-life care conjunction with other issues affecting carers. This review set out to synthesise the findings of these prior qualitative studies, in order to examine common themes regarding carers’ experiences and perceptions of end-of-life with a long-term neurological condition.

## Methods

In line with institutional procedures, the [Institution Name] Ethics Committee was notified that this review was taking place on 7th March 2019.

Meta-ethnography is a systematic approach to the interpretative synthesis of qualitative evidence.^15–17^ Rather than seeking solely to summarise studies, meta-ethnography seeks to translate the findings from studies into each other, in order to build upon the qualitative characteristics of the original studies and to generate new theoretical knowledge. Meta-ethnography was chosen as an approach that would preserve the qualitative character of the original studies exploring end-of-life care. Noblit and Hare set out seven steps for meta-ethnography, which this study followed:

1. *Getting started*: The original idea for the study arose from an earlier local research project to explore neurological needs, which identified that carers and family members of people with long-term neurological conditions experienced challenges around end of life. [REFERENCE OMITTED FOR PEER REVIEW]. This study therefore set out to review the experiences and perceptions of informal carers of adults living with long-term neurological conditions regarding end of life.
2. *Deciding what is relevant to the initial interest*: The study sought to include all recent qualitative studies relating to informal carers’ experiences of end-of-life for adults with a long-term neurological condition. In devising a search strategy, the authors were conscious that there are several hundred neurological conditions, some of which are unlikely to be discussed in an end of life context (e.g. migraine). The search strategy therefore incorporated both the generic term ‘neurological’, and the names of specific long-term conditions where the previous research project suggested that there was existing palliative care literature. Papers focused on primary dementia (e.g. Alzheimers) were excluded on the basis that dementia is the subject of a relatively large, distinct body of caregiving literature.

In order to get a range of literature from medical, health and social science perspectives, the following databases were searched: Academic Search Complete, Allied and Complementary Medicine Database, Applied Social Sciences Index and abstracts, CINAHL Complete, MEDLINE, PsycINFO, Scopus, Soc Index, Web of Science. The search was initially carried out in November 2018, and updated June 2020.

The search was an abstract search for the following search strings, adapted as appropriate to the search engines for each database.

Search String 1: carer or caregiver
Search String 2: Neurological or Parkinsons or Stroke or Multiple Sclerosis or Huntington’s Disease or Motor Neurone Disease or Amyotrophic Lateral Sclerosis or Traumatic Brain Injury or Post-Polio Syndrome or Muscular Dystrophy or Multiple System Atrophy
Search string 3: End of life or palliative or bereavement or dying or death or hospice

Limiter applied within the database: Published after 1 January 2010.

Inclusion criteria were that papers were English language, published since 2010, and based upon qualitative data collection and analysis from informal carers of adults living with neurological conditions. All or part of the research findings/analysis were required to address carers’ experiences or perspectives related to end of life, palliative care, dying and/or bereavement. Papers were excluded if they did not address carers’ perspectives; related solely to carers of children or of people with dementia; or if in- and out-of-scope material was grouped together in a way that did not allow for separate analysis (Table 1).

**Table 1.** PRISMA flowchart.

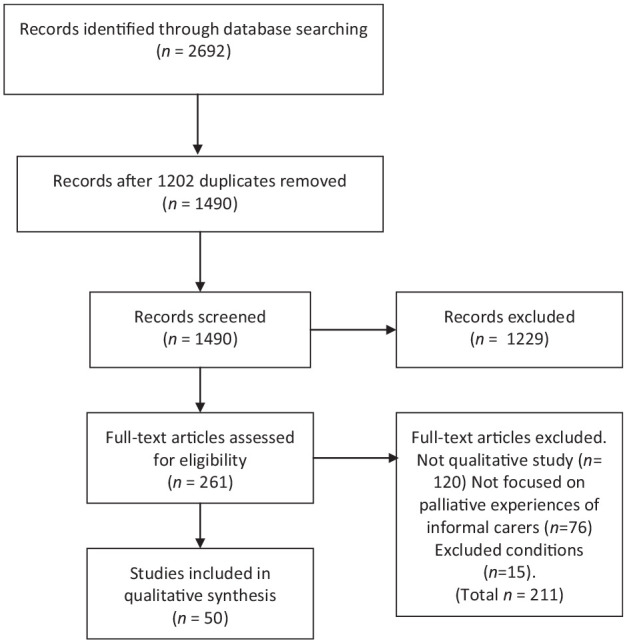

Abstract screening was undertaken by MT, with a random sample of 10% independently screened by MR to ensure inter-rater reliability. In line with other meta-ethnographic studies, it was often unclear at abstract screening whether papers met the inclusion criteria.^17^ On full-text screening, a relatively high proportion ultimately proved to either not be qualitative research, or to not be focused upon the perspective of carers, and were excluded. Where papers included reference to carers’ perspectives on end-of-life, but this was not the sole focus of the paper, both authors discussed and agreed whether there was sufficient relevant material to include. Where there was more than one paper from the same study or sample, all were included, as they often addressed different aspects of end-of-life. However, this was considered in the analysis stages to avoid giving undue weight to multiple papers from the same study.

An assessment of study quality was not undertaken in deciding which studies to include or exclude. There are differing perspectives on the value of quality assessment in meta-ethnography, and even where quality assessment is used, reviewers do not necessarily exclude low-scored studies.^17–19^ Quality assessment of published qualitative research may be affected by discipline and journal, and may tend to be affected by word count limitations.^17,20^ Methodology and conceptual framing may ultimately be more relevant than quality in attempting to translate and synthesise studies.^17^ In the latter stages of synthesis, we aimed to reflexively and rigorously consider the different methodologies and focus, and the possible consequences for our analysis

3. *Reading the studies*: Both authors read and re-read the selected articles to familiarise ourselves with the research studies’ context and focus, and their conclusions about carers’ experiences of end of life. Basic data about study design, setting and sample was extracted into a Table 2.
4. *Determining how the studies are related.* Initially, both authors independently read papers and identified key themes and supporting evidence. This was done through close repeated reading of the entire paper. We used a grid to extract the key themes developed by the original author(s), example quotations from study participants (if present), and our own reflective notes on the papers.
5. *Translating the studies into one another.* The authors discussed our analyses between ourselves and inductively identified what we considered to be the key overarching themes, through grouping related concepts extracted from the papers, with repeated reference back to the original text to ensure that meaning was not lost, and that core themes identified by the original study authors were captured in our over-arching themes.

**Table 2.** List of included studies.

Study author	Country	Research question/aims	Design	Setting/participants	Data collection	Findings (themes)
Anderson et al.^33^	Australia	‘This study aimed to understand caregiving experiences in MND [motor neuron disease], to make recommendations regarding the development of support interventions’	Qualitative thematic analysis, informed by realist perspective	About 15 caregivers of people living with motor neuron disease, recruited from a neurological clinic. 14 spouses, 1 adult child.	Semi-structured interviews	1. The Thief (experience of loss and grief across varied facets of life) 2. The Labyrinth (experience of finding ways to address the ever-changing challenges as the disease progressed) 3. Defying fate (experience of resilience and hope as caregivers tried to make the most of the time remaining with their loved one)
Aoun et al.^48^	Australia	‘This study explored the experiences of MND [motor neuron disease] family carers, both during their time as carers and following bereavement. Particular attention was paid to the carers’ prolonged grief status and to the implications for service delivery, including palliative care’	Qualitative thematic analysis	About 16 former spousal-carers of people with motor neuron disease, recruited via community organisation 1–4 years after spouse’s death	Semi-structured interviews	1. The work of family carers 2. The change in relationship from spouse to family carer 3. Family caring as a series of losses 4. Coping mechanisms of family carers 5. Supportive and palliative care experiences of family carers
**Aoun, Deas et al**.^67^	Australia	‘Our aim was to assess the feasibility and relevance of the CSNAT [carer support needs assessment tool] in home-based care during the caregiving period from the perspectives of the family caregivers of people with MND [motor neuron disease] and their service providers’	Descriptive longitudinal study assessing intervention	About 24 family caregivers (19 spouses, 3 parents, 1 adult child, 1 sibling), recruited from clients of motor neuron disease support organisation.	Semi-structured interview	1. The overwhelming caregiver journey with motor neuron disease 2. Tool practicality and usefulness; 3. Validation of the caregiver role and empowerment; and 4. Reassurance of support.
**Appleton et al.** ^47^	N/A – no geographic limits applied	‘The aim of the synthesis was to focus on spousal/partner carers of people with MS and their experience of caring and their caring relationship, to enable a conceptually richer understanding of the experience of being a carer, and better inform the provision and development of services’	Qualitative meta-synthesis (meta-ethnography)	20 papers	Systematic review and literature synthesis	1. Acceptance and appreciation 2. Commitment 3. Setbacks with services 4. Shifting sands 5. Becoming the carer 6. Living with loss.
Baxter et al.^43^	England	‘The purpose of the present study was to investigate the experiences of family carers and health care professionals who were involved in caring for patients with MND [motor neuron disease] using NIV [non-invasive ventilation]. We aimed to explore attitudes and perceptions regarding use of NIV at the end of life’	Qualitative longitudinal study, thematic analysis	Nine carers of people with MND recruited via neurology clinics. Carers recruited on basis of relationship to an eligible patient (diagnosis of motor neuron disease, required non-invasive ventilation and had since died)	Semi-structured interviews.	1. Unexpected speed of deterioration 2. Hospitalisation versus dying at home 3. Attempts to resuscitate 4. Decision-making regarding the withdrawal of non-invasive ventilation 5. Peaceful final moments 6. Turning off the machine 7. Professional uncertainty regarding the use of non-invasive ventilation 8. Positive impacts of non-invasive ventilation use 9. Concerns regarding non-invasive ventilation use
Bentley and O’Connor^50^	Australia	‘This study examined the perceptions of EOL [end-of-life] experiences of family carers of people with MND [motor neuron disease] in Western Australia to identify unmet needs and gaps in EOL support for people with MND and their family carers’	Descriptive qualitative study using a social constructivist framework and thematic analysis	About 12 carers (11 spouse, 1 child) of people with motor neuron disease, recruited via community organisation, 3–15 months after person’s death	Semi-structured interviews.	1. Accessing appropriate supports, 2. Accessing information 3. Feeling prepared.
Boersma et al.^5^	US	The objective of the present study was to validate and build on [prior research] by eliciting Parkinsons Disease caregiver needs, salient concerns, and care preferences.	Open-ended qualitative analysis, within a broader study.	About 15 caregivers of people with Parkinsons, recruited via medical service and community group	Semi-structured interviews (11 participants) and focus group (4)	1. The need for individual attention and support; 2. Educational needs and helpful resources; 3. The consequences of motor and nonmotor symptoms; 4. Concerns about the future; 5. Responses to palliative care.
Bowen et al.^26^	UK	‘What are the psychosocial and relationship needs of family members when adapting to a relative’s MS [multiple sclerosis]? In particular, how do these needs change or develop in the context of disease progression when the family member with MS (fmwMS) is admitted into care as resident or for respite?’	Qualitative, grounded theory	About 25 relatives (5 parents, 7 spouses, 5 siblings and 8 children) of people with MS at seven care centres	Semi-structured interviews	1. Information, communication and understanding 2. Family relationships, roles and responsibilities 3. Emotion, coping and support 4. Life, outlook and reflection
Cipolletta and Amicucci^24^	Italy	‘The aim of this study was to explore the experience of family members who live with ALS patients until their death’	Qualitative, Interpretive phenomenological analysis	About 13 participants (8 children, 4 partners, 1 sibling) recruited via online support forum.	Semi-structured interviews.	1. Meaning of ALS 2. Family relationships 3. Health care context.
Davies et al.^51^	Wales	‘In this study, we aimed to explore the experiences of pwMS (people with Multiple Sclerosis) of transition from relapsing onset MS to SPMS (Secondary Progressive Multiple Sclerosis) and to document views of carers during this period’	Qualitative design based on social constructionism, thematic analysis	About 14 carers (8 spouses, 4 parents, 1 sibling, 1 friend), recruited via patients listed on a hospital MS database	Semi-structured interviews	1. Finding out about the transition. 2. What secondary progressive disease means 3. Living with progressive disease
Ebrahimi et al.^6^	Iran	‘. . .this study’s main goal [is] to highlight, recognise and explain the family experiences of MS [multiple sclerosis] patients and problems they face in Iran’	Qualitative study, content analysis	About 18 carers (12 spouses, 5 mothers, 1 daughter) of people with MS, recruited via hospital	Semi-structured interviews.	1. Disease onset crisis 2. Disease burden 3. Living in the shadow of death.
Flemming et al.^34^	N/A – no geographical limiters	‘To explore the experiences of, and need for, palliative care for adult patients with motor neuron disease and their informal carers across the disease trajectory, through a systematic review of qualitative research’	Qualitative systemative review	About 41 papers were included, giving the experiences of 369 caregivers	Thematic synthesis	1. Response to diagnosis 2. Maintaining control 3. Deterioration and decision-making 4. A life of unremitting loss 5. Engaging with professional support 6. Planning for end of life 7. Carers’ experiences of end-of-life care 8. Bereavement
Fox et al.^7^	Republic of Ireland	‘The primary aim was to explore the palliative care and related issues affecting PwPD [people with Parkinsons’ Disease] and their families. A secondary aim was to explore participants’ perceptions of palliative care and elicit potential barriers or facilitators to accessing or receiving this care’	Qualitative, analysed according to consolidated criteria for reporting qualitative research	About 12 carers (9 spouses, 2 siblings, 1 child) of people with Parkinsons recruited via 3 movement disorder clinics	Semi-structured interviews	1. Patient burden 2. Carer burden 3. Information and support needs: no ‘one-size fits- all’ 4. Crisis at diagnosis. 5. Experience of healthcare services: feeling unsupported 6. Advance care planning. 7. Perceptions of palliative care.
Francis et al.^35^	Denmark	‘The aim is to explore ethical dilemmas that spouses experience in the everyday care of a partner in treatment for PMBT [primary malignant brain tumour]’	Qualitative descriptive	About 10 spousal carers, recruited via a hospital clinic	Semi-structured interviews	1. Doing the right thing in unpredictable daily situations, 2. Torn between patience and guilt 3. Living in a time of uncertainty, hope and despair.
Freer^27^	UK	‘The aim of this research was to capture carers’ experience of caring for a family member with MND [motor neuron disease]’	Qualitative, descriptive, thematic analysis	Nine carers (8 spouses, 1 daughter-in-law) recruited via an MND outpatient clinic	Semi-structured interviews.	1. Motor neuron disease (MND): the specifics and the differences 2. Normality versus reality 3. Defining the total loss but holding onto hope 4. Information needs and support—at whose pace?
Giovannetti et al.^66^	Italy	‘We performed a qualitative study to investigate the experiences of participants in a multicentre randomised controlled trial on a home-based palliative approach (HPA) for adults with severe multiple sclerosis (MS) and their caregivers. Our aim was to explore the strengths and challenges of the intervention, and circumstances that may have influenced its efficacy’	Qualitative framework analysis, nested in a mixed methods study.	About 15 carers (6 spouses, 3 parents, 3 children, 3 other) recruited from those receiving a pilot intervention for Multiple Sclerosis at three centres	Semi-structured interviews and focus groups	1. Expectations 2. Met and unmet needs 3. Barriers
Gofton et al.^28^	Canada	This study aimed to develop a conceptual understanding of the specific characteristics of palliative care in neurology and the challenges of providing palliative care in the setting of neurological illness.	Qualitative, grounded theory	Two patient-carer dyad interviews, 1 carer interview, recruited via a hospital centre	Semi-structured interview	1. Timelines of disease progression, 2. Barriers to communication arising from neurologic disease, 3. Variability across disease progression, 4. Threat to personhood arising from functional and cognitive impairments related to neurologic disease. 5. Uncertainty with respect to prognosis, support availability and disease trajectory 6. Inconsistency in information, attitudes and skills among care providers, care teams, caregivers and families. 7. Existential distress specific to neurological disease, including emotional, psychological and spiritual distress resulting from loss of function, autonomy and death.
Golla et al.^49^	Germany	‘Our aim was to gain an insight into the subjectively unmet needs of caregivers of severely affected MS [multiple sclerosis] patients in Germany’	Qualitative content analysis	About 12 carers (6 parents, 3 partners, 1 sister, 1 daughter, 1 friend) of people with multiple sclerosis, recruited via self-help groups, nursing home, hospital and community volunteer	Semi-structured interview.	1. Relationship to physician 2. Individual support by the healthcare system 3. Relationship to the individual severely affected by MS 4. End-of-life issues 5. Self-care 6. Higher awareness of multiple sclerosis
Halpin^41^	US	The article aims to ‘provid[e] the first completely qualitative study of suicidality in the HD [Huntington Disease] community’.	Qualitative, grounded theory	About 10 caregivers (9 spouses, 1 grandparent).	Semi-structured interview.	1. The perspective of informal caregivers 2. Suicidality in Huntington Disease Families 3. Personal accounts of suicidal behaviour.
Harris et al.^64^	England	‘This study aims to extend the literature by exploring the meaning of supporting a loved one dying with MND [motor neuron disease], so it can enhance nurses’ and other healthcare professionals’ understanding of dying for this client group’	Autoethnography	One participant (author, daughter of person with motor neuron disease)	Autoethnographic	1. Loss of person (lived body experienced in silence); 2. Loss of relationships (lived relations are challenged); 3. Loss of home and loss of time (lived space and lived time take on new meaning); 4. Loss of future (dying-facing it alone)
Hasson et al.^10^	Northern Ireland	‘This study aims to understand the experiences of family carers who cared for someone with Parkinson’s disease so that their role might be recognised and supported’.	Qualitative, exploratory descriptive design, thematic analysis	About 15 former caregivers (10 spousal, 5 child) of people with Parkinsons, recruited via support groups and public posters	Semi-structured interviews	1. Carers’ role and burden 2. Palliative care 3. Bereavement 4. Access to health and social care services.
Kavanaugh et al.^61^	USA	The purpose of this study is to provide an initial exploration into what caregiving youth know about their parent’s advance care planning and their experiences with having EOL [end of life] discussions with the parent.	Mixed methods, within a larger study. Qualitative element was thematic content analysis	About 40 adolescents aged 12–20, engaging in some caregiving activity for a parent with Huntington’s, recruited via community group. 24 out of the overall sample discussed end of life.	Semi-structured interviews	1. Respect for the parent’s EOL wishes 2. Caregiving youth’s opinion not valued 3. Avoidance of EOL issues 4. Protect the parent 5. Parent in denial 6. Parent not ready to have conversation 7. Realisation of the terminal outcome
Lerum et al.^25^	Norway	‘This paper explores the meaning of chronicity and terminality in motor neuron disease (MND), also known as amyotrophic lateral sclerosis (ALS)’	Qualitative, thematic analysis	Eight bereaved carers and 17 current carers of people with MND, recruited via three hospital sites	Narrative interviews.	1. The trajectory 2. The social subworlds 3. Instability and prognosis in the hospital 4. Prognostic dependency in primary care 5. Unstable terminality at home
Lerum et al.^25^	Norway	‘We conducted a qualitative study to understand more about family caregivers’ work and sense of responsibility, exploring family caregivers’ accounts of caring for a family member with MND’	Qualitative, thematic analysis	Eight bereaved carers and 17 current carers of people with MND, recruited via three hospital sites	Narrative interviews.	1. Immediate care work 2. Seeking information and clarity 3. Managing competing obligations 4. Maintaining normality 5. Managing external resources and assistance
Lu et al.^36^	China	‘The aim of this study was to explore the experience of family caregivers taking care of stroke survivors in China’	Qualitative, explorative design	About 26 carers (21 spouses, 4 adult children, 1 daughter-in-law)	Semi-structured interviews	1. Living on the edge 2. Having total responsibility 3. Being all alone 4. Drained by caregiving 5. Being a prisoner in their own life 6. Being uncertain about the future
Mantell^63^	UK	‘1. What are the common and significant experiences for relatives in adapting to caring for a person with HD? 2. How are these experiences affected by support from others, including formal service provision?’	Qualitative, grounded theory	About 31 carers (18 spouses, 4 parents, 7 children, 2 siblings, 2 in-laws, 5 cared for multiple relatives) of people with Huntingtons	Semi-structured interview.	1. The journey to diagnosis 2. Changing roles, changing relationships 3. Care- centric relationships 4. Power and dependency 5. Redefining care 6. Behind the mask of Huntingtons Disease 7. Redefining the relationship 8. Entering care homes 9. Loss and bereavement
McCurry^62^	US	‘The aim of this study was to examine decision making by informal caregivers of MS [multiple sclerosis] care recipients. Specifically, the researcher sought to understand what types of decisions the caregivers were making and what resources they used to make those decisions’	Qualitative, thematic analysis	Six carers (4 spouses, 1 fiancee, 1 friend) of people with MS, recruited via a hospital centre and support group	Two in-depth interviews with each participant.	1. Healthcare 2. Financial 3. Social 4. Family
McLaughlin et al.^59^	Northern Ireland	‘This study, therefore, set out to explore the experience of informal carers of people with PD [Parkinsons Disease]’	Exploratory qualitative study, framework analysis	About 26 spousal carers for people with Parkinson’s, recruited via support groups and public posters	Semi-structured interview,	1. Medical support for people with Parkinsons Disease 2. Burden related to care giving, 3. Information needs 4. Economic implications of caring.
Mc Veigh et al.^58^	Northern Ireland	‘To explore the provision of generalist and specialist palliative care in Northern Ireland, at the end of life, for people with MND [Motor Neuron Disease] from the perspective of bereaved carers’	Exploratory interpretivist approach	About 13 bereaved carers (9 spousal, 3 daughters, 1 grandson), identified through Motor Neuron Disease register.	Semi-structured interviews	1. The provision of holistic care 2. The biopsychosocial impact of MND 3. Lack of death preparedness.
Murray et al.^40^	Australia	‘We aimed to investigate caregiver perspectives on the acceptability and impact of advance care planning, documented in a letter format, for patients with motor neuron disease and caregivers’	Qualitative cross-sectional study, narrative synthesis	About 18 former carers (15 spouses, 2 children, 1 sibling) of someone who had died from Motor Neuron Disease, recruited via a hospital service	Semi-structured interviews.	1. Readiness for death 2. Empowerment 3. Connections 4. Clarifying decisions and choices
Mutch^29^	England	‘To gain a deeper understanding of the experiences of the partner living with and caring for a spouse disabled by multiple sclerosis’	Qualitative study, thematic analysis	Eight spousal carers of people with MS under the care of a neurologist at a specific centre	Semi-structured interviews	1. Worry 2. Planning 3. Frustrations 4. Commitment to marriage 5. Coping strategies
O’Brien and Preston^21^	England	To explore ‘the experience of hospitalisation following a diagnosis of motor neuron disease from the perspective of family carers of those diagnosed with the illness’	Qualitative secondary analysis of data collected in two previous qualitative studies (cited here as Preston and Whitehead and O’Brien et al.)	About 18 bereaved carers and 3 current carers (all spouses except one son and one father and son).	Narrative and semi-structured interviews.	1. Lack of knowledge 2. Basic care 3. Reluctance for admission 4. Final memories.
O’Brien et al.^23^	England	‘To explore, from a qualitative perspective, the views of current and former family carers of people with MND and identify their need for, and use of, support services’	Thematic framework approach	About 18 current carers and 10 bereaved former carers of people with Motor Neuron Disease, on the database of a care and research unit	Narrative interview	1. The impact of being a carer 2. The provision of information 3. Experiences with paid-for in-home care, 4. Respite care 5. The need for counselling 6. Carers’ training needs.
O’Connor et al.^57^	Australia	‘The aim of the study was to describe the experiences of family carers of people with MND [Motor Neuron Disease] in receiving the diagnosis in order to inform and improve ways in which the diagnosis is communicated’	Qualitative thematic analysis	About 190 carers of people with Motor Neuron Disease, surveyed via community organisation	Cross-sectional survey	1. Frustrations with the diagnosis 2. Giving information 3. Family carer observations of the neurologist 4. The setting 5. What would have made the diagnosis easier
Oyebode et al.^30^	UK	‘This study aimed to provide an in-depth qualitative exploration of the experience of living with, and caring for, a partner with MND [motor neuron disease]’	Interpretive phenomenological	Eight spouses/partners of people with Motor Neuron Disease, recruited via Motor Neuron Disease centre	Semi-structured interviews.	1. impact on life 2. Adjusting to the situation
Ozanne et al.^31^	Sweden	‘The aim of this study was to illuminate experiences of finding meaning in life among spouses of people with amyotrophic lateral sclerosis’	Qualitative content analysis	About 13 spouses/partners of people living with amyotrophic lateral sclerosis and using a particular hospital service.	Semi-structured interview.	1. Feeling limited and isolated in the proximity of death 2. Finding meaning despite the proximity of death
Payne et al.^54^	England	‘The aims of this qualitative study were to identify patients’ and family members’ experiences of acute stroke and their preferences for end-of-life care’	Cross-sectional qualitative exploratory design.	About 25 family members of stroke patients (45% spouses), hospitalised in a particular unit	Semi-structured interview.	1. Communication and information provision 2. Facing uncertainty and end-of-life preferences
Penrod et al.^46^	US	‘The purpose of this study is to illustrate variations in caregiving trajectories as described by informal family caregivers providing end-of-life care’	Instrumental case study, grounded theory	About 46 people who have provided end of life care, of whom 10% were caring for people with amyotrophic lateral sclerosis. Article focuses in depth on one case study relating to amyotrophic lateral sclerosis.	Unstructured interviews	1. Sensing a disruption 2. Challenging normal 3. Building a new normal 4. Reinventing normal.
Preston et al.^22^	England	‘This study aimed to look at MND [motor neuron disease] patients’ bereaved relatives’ experiences of using the PPC [preferred priorities for care] document and their perceptions about its impact on end-of-life care’	Qualitative thematic analysis	About 11 relatives or carers of people with MND, on the database of MND care and research unit	Semi-structured interviews	1. Completion; 2. Document availability to others; 3. Importance and influence on the end-of-life experience; 4. Limitations.
Rademeyer et al.^37^	New Zealand	‘The aim of this study was to explore the phenomenon of becoming and living as a family over the first 2 years following a family member’s first-time stroke’	Hermeneutic enquiry	Four family members of one stroke survivor (husband, daughter, son and daughter’s partner), identified from hospital	Semi-structured interviews (3 with each participant)	1. Experiencing shocks during life and death 2. Unconditional devotion 3. Continual change.
Ray et al.^42^	England and Australia	‘This study sought to examine the different ways family caregivers of people living with MND [motor neuron disease], constructed dying and the death event of their relative. Given the neurologically progressive, degenerative nature of MND, we wanted to know about their planning for and experiences of dying and death, to enable health and social practitioners to develop better interventions to support family caregivers providing end-of-life care’	Secondary thematic analysis of qualitative data	About 18 caregivers of people with motor neuron disease from Australia and 11 from England (all partners except for one daughter), recruited via community orgs and interviewed after patient’s death	Semi-structured interviews,	1. Planning for end of life 2. Unexpected dying 3. Dignity in the dying body 4. Positive end to MND
Stavroulakis et al.^32^	UK	‘The aim of this study was therefore to explore the decision-making process in relation to timing of gastrostomy insertion from the perspective of the patients [with motor neuron disease] and their informal carers’	Qualitative approach, thematic analysis	Eight carers of people with motor neuron disease who received gastrostomy at a hospital centre	Semi-structured interview	1. Prolonged, tiring and effortful meals 2. The task of food preparation 3. Choking and aspiration 4. Weight loss. 5. Reluctance to give up oral feeding, 6. Uncertainty over disease trajectory 7. Not realising the potential benefits 8. Negative perceptions of gastrostomy
Veronese et al.^39^	Italy	‘This study aimed to look at the needs of people with neurodegenerative disease in the Turn area and assess how they would see a specialist palliative care service helping them’	Qualitative observational study, content analysis	About 21 carers of people with neurological conditions (main carer spouse in 19 cases), whose family member had been referred into the study by specialists	Interviews	1. Physical issues 2. Psychological issues 3. Social issues 4. Spiritual issues
Veronese et al.^39^	Italy	‘The study aimed to identify how the decision of a tracheostomy was taken by the patients, and collect information from family carers about the end of life phase of ALS [amyotrophic lateral sclerosis] patients who died after being tracheotomised and mechanically ventilated, looking in particular at the possibility of the prediction of end of life in these patients and the possible involvement of specialist palliative care’	Qualitative study, content analysis	About 19 carers (11 spouses, 7 children, 1 long-term live in paid carer) of people with amyotrophic lateral sclerosis who had received a tracheostomy and since died (random sample)	Semi-structured interviews.	1. The process of consent to the tracheostomy 2. The predictability of deterioration at the end of life.
Wallengren et al.^55^	Sweden	‘The aim of this study is to explore relatives’ information needs and the characteristics of their information-seeking process for the day of the stroke to the end of the subsequent 6-month period’	Qualitative study with descriptive design	About 16 relatives of stroke survivors (6 spouses, 8 daughters, 1 daughter-in-law, 1 friend) admitted to stroke unit	Open ended interview, content analysis	1. Information needs 2. Characteristics of information seeking.
Warrier et al.^38^	India	‘The current study aimed at understanding the lived experiences of spouses of persons with MND [motor neuron disease] in India’	Qualitative inquiry, interpretive phenomenological analysis	Two spousal caregivers.	Semi-structured interviews (3 with each participant)	1. Meaning of motor neuron disease 2. Relationship 3. Adaptation 4. Life without the loved one
Warrier et al.^38^	India	‘This study explored, (a) the caregivers’ experiences of the end-of-life stage, and (b) the sources of support for individuals with MND [motor neuron disease] at the end-of-life stage and their caregivers. An attempt was made to understand ‘what it is like to be a family caregiver during the death and bereavement of a person with motor neuron disease’	Qualitative exploratory study, thematic analysis	Seven bereaved carers (6 spouses, 1 son) of individuals with motor neuron disease, recruited from national tertiary care centre.	Semi-structured interviews	1.Transition from person to patient 2. Support during advance stages 3. Death 4. Impact on the caregivers.
Weisser et al.^65^	UK	‘The aim of this study was to explore in depth the experiences of a subgroup of carers of people with MND/ALS [motor neuron disease/amyotrophic lateral sclerosis], specifically the relationship between positive and negative experiences of caring, and to identify possible ways to better support them’	Qualitative secondary thematic analysis	About 10 spouse/partner carers of people with motor neuron disease/amyotrophic lateral sclerosis, recruited via community groups and neurology clinics	Semi-structured interviews (2 or 3 per participant)	1. Resilience (positive/active) 2. Being rewarded (positive/passive) 3. Carrying a burden (negative/active) 4. Having needs (negative/passive)
Whitehead et al.^56^	England	‘To explore the experiences of people with Motor Neuron Disease, current and bereaved carers in the final stages of the disease and bereavement period’	Qualitative phenomenological approach, thematic analysis	About 18 current carers and 10 bereaved former carers of people with motor neuron disease, on the database of care and research unit	Narrative interview	1. Anxieties 2. End of life decision-making and advance care planning 3. Services at the end-of-life stage 4. Impact on carers 5. Euthanasia
Winther et al.^52^	Denmark	‘The aim of the study was to explore everyday life experience of relatives of people with ALS [amyotrophic lateral sclerosis] living at home with mechanical ventilation and formal caregivers’	Qualitative phenomenological-hermeneutic approach	Nine current and two bereaved relatives, recruited via a respiratory centre. Six were spouses, two were former spouses, along with one brother, one daughter and one sister-in-law	Semi-structured interviews	1. We are in this together until the end 2. Vulnerable relatives fighting to keep track of everything 3. Formal caregivers—a distressing relief 4. A prison without personal space

Because some papers were not solely focused upon carers and end-of-life, some themes identified within the papers were excluded from this review, such as those solely relating to patient or practitioner experiences. Other studies addressed the overall experience of caring, from pre-diagnosis to death. In studies of care for life-limiting illness, it is difficult, and perhaps somewhat artificial, to wholly distinguish between end-of-life and the broader context of care. However, upon discussion, and in order to preserve the focus on end-of-life, we decided to exclude analysis that seemed to be predominantly focused upon more general experiences of being a carer (e.g. financial strain), rather than upon anticipating or experiencing end of life.

6. *Synthesising the translations.* Noblit and Hare suggest that studies that are on very closely related topics may reciprocate or refute each other, while studies that do not overlap so closely may point to an overall ‘line of argument’ addressing a bigger picture than the individual studies address directly.^15^ While few papers featured all of the five themes identified, across the spread of papers there appeared to be a largely consistent line of argument as to what it was to be a carer of a person with a neurological condition approaching the end of life.
7. *Expressing the synthesis.* This paper constitutes the expression of synthesis, and was written jointly between the two authors.

## Results

### Study characteristics

In total, 50 papers were included (see Table 2). However, in some cases there was more than one paper from the same data collection, and other papers utilised overlapping samples.^21–23^ Papers were predominantly based on data from North America, Australia or Western Europe, with participants typically recruited from hospitals, support groups and research databases. In some cases, recruitment of carers had been secondary to recruiting patient participants for other workstreams. Where relationship between carer and care recipient was specified, the majority were spouses or intimate partners. Most papers utilised semi-structured interviews.

Most papers were specific to one neurological condition, with 27 out of 50 focusing on Motor Neurone Disease/ Amyotrophic Lateral Sclerosis. This does not reflect the prevalence of conditions. Motor Neurone Disease is less prevalent and makes less of a contribution to global burden of disease than conditions such as stroke, Multiple Sclerosis and Parkinson’s,^1^ suggesting that carer experiences of some common neurological conditions may be comparatively less well researched.

Five overarching themes were identified as set out at Table 3.

**Table 3.** Key Themes.

Themes	Key concepts within theme
Future (un)certainties	Certainty of death
	Uncertainty over speed of progression, symptom progression
Information paradox	Diversity of information needs
	Importance of sensitive and timely conveyance of information
Access to support	Barriers to accessing support
	Medicalisation of support
	Lack of access to emotional support (including bereavement
Taking decisions	Desire for loved one to take own decisions (but often being expected to do so)
	Decisions being overturned by professionals
Continuity, change and disruption	Maintaining a sense of normality and challenges to that
	Emotional commitment
	Loss and bereavement

### Future (un)certainties

Underpinning the accounts of informal carers were questions of certainty and uncertainty with regard to the progression and prognosis of neurological conditions and the proximity of death. Particularly within the papers focusing on Motor Neurone Disease, and advanced Multiple Sclerosis, carers were distressed and frustrated at a situation where they perceived their loved ones to have received a ‘death sentence’ (p. 290)^24^ or to be ‘living in the shadow of death’ (p. 16),^6^ yet simultaneously facing substantial uncertainty over when death would occur and what would happen in the meantime, resulting in a feeling of living in ‘limbo’ (p. 91).^25–38^ Some papers highlighted lack of certainty about the future deriving from lack of knowledge: for example, Fox reports that some carers were not aware that Parkinson’s was incurable.^7^ A small number of papers reported cases where the carers and the person they cared for did not accept the certainty of death, for example, pursuing experimental treatments overseas.^39,40^ Among carers of people living with Huntington’s, the certainty of a degenerative life-limiting condition was seen as particularly difficult to bear, with suicide sometimes perceived as an understandable result.^41^

Even within the context of a life-limiting condition, death itself was sometimes unexpected. Some papers reported that carers had anticipated a gradual decline, and were surprised by what they perceived as a sudden death.^42–44^ However, Veronese et al. suggested that carers often were aware from identifiable features that death was near.^45^ The uncertainty entailed within neurological conditions was also sometimes contrasted to other conditions, although not consistently. Penrod used Amyotrophic Lateral Sclerosis as an example of an expected death trajectory, specifically contrasted to cancer and heart failure as mixed or unexpected death trajectories.^46^ However, Gofton et al. argued that within palliative care, timelines for progression of neurological disorders are longer and more uncertain than those of oncology.^28^ They highlight that uncertainty arises in several dimensions, including diagnosis, prognosis and disease trajectory, and is interrelated with inconsistency in information and support, and underlying existential distress from the situation itself.

### Information paradox

While giving information to families at the time of diagnosis is crucial, studies highlighted that information-giving was inconsistent and at times inadequate.^7,26,47^ Carers gave examples of professionals sharing diagnosis in an insensitive manner, leading to a perception that the individual and family consequences of diagnosis were neither recognised nor addressed.^7,24,34,48,49^ Carer experience of poor, absent or delayed information about support services and resources resulted in feelings of isolation and additional strain.^34,47,50^

Despite the agreed importance of sharing information about end of life, carers reported difficulties in accessing information. At times this was due to variable practice in talking about end of life and related topics such as advanced planning and treatment options.^42^ Carers were also often unsure who, from a multitude of professionals, they should approach to discuss end of life.^7,23,24,50–52^ Confusion about responsibility for sharing information about end of life could be exacerbated by factors such as poorly integrated services, lack of confidence amongst professionals and a lack of clarity about what had already been shared.^50^

Paradoxically, some studies also highlighted risks associated with being given excessive information, such as information that was unwanted, poorly-timed, or poorly-delivered.^53^ Factors such as culture, coping mechanisms and within-family relationships influence the kinds of information that families need and how they prefer it to be given.^26,27^ Carers have diverse information needs, which may change over time,^54,55^ which has significant implications for the ongoing assessment of carer information needs.

### Access to support

Carers experienced a number of barriers in accessing support services and the identification of support needs was often overlooked by HCPs. Carers often experienced accessing services as longwinded or disjointed, especially when managing the process at critical points in their relative’s illness and care trajectory.^7,10,48–50,52,56^ The point of diagnosis was frequently cited as a moment where carers would have valued additional emotional and practical support.^10,34,48,49,57^ Through the course of long-term neurological conditions, families/carers consistently cited difficulties in accessing a range of support including, for example, infrequent contact with specialist clinics and opportunities for families to talk with professionals in depth;^7,26,50,51^ a lack of appropriate care services,^23,36,56^ a lack of a whole family approach to support^26^ and poor access to palliative or specialist support services.^5,7,26,47,50^ Although specialist services were generally appreciated, as conditions progressed, carers and families sometimes felt isolated by a focus on symptom management at the expense of emotional, psychological and social support.^24,51,58^ Perceptions of ad hoc or poor communication meant that families were not always aware of potential support services until a crisis occurred or situations had deteriorated.^23,56,59^ Some families that required help found it difficult to accept help when offered or did not know what kind of support might be helpful.^7,23,26,39,48,51^ When family members began receiving formal care, the change in role for carers also at times caused tensions.^37,52^

Access to palliative care frequently occurred late in the illness trajectory.^48,50,59^ Poor awareness of palliative care services and frequent misperceptions that they were only for people who were dying or for people who had cancer, served as barriers to proactively accessing palliative care.^7,10,50,59^ If, or when, palliative care was received, carer/family member experiences were generally positive about their experience but, overall, improvements in communicating the role and purpose of palliative care combined with timely access to those services could contribute to supporting carers at a time of great stress.^10,48,50,56,58^ However, Aoun reported that an intervention offering dedicated support assessments for carers was welcomed.^60^

The absence of bereavement support was notable, as was the experience of being suddenly cut off, following the death of their relative, from an often extensive network of health and social care professionals.^10,34,48,56^ Concerns for carers being overwhelmed by the stress of providing care, followed by the death of their relative, were cited as possible risk factors for complex and prolonged grief.^23,48^

### Taking decisions

Carers’ experiences of end-of-life often involved decisions on behalf of their loved one, for example with regard to ventilation or gastrostomy, or supporting the drawing up of advanced care directives. A number of papers in this review specifically focused upon such decisions. Carers typically wished their loved one to take key decisions themselves, yet in practice this did not always occur.^22,40,43,56,61^ Sometimes, the progression of neurological conditions meant that the care recipient was less able to take or communicate decisions, requiring the carer to take a greater role.^5,21,22^ Ray highlighted that some care recipients did not want to discuss end of life.^42^ In other cases, the urgency of situations overtook decisions.^25,40,43,58^ At times, carers were asked to overturn decisions their loved one had already made, for example to accede to a professional’s views on attempting resuscitation.^34,40,42,45,58^ Documentation of decision making was perceived as helpful, but carers reported an ongoing need for input to ensure that documented decisions were implemented.^22,25,40,56^

Some papers highlighted barriers to carers being involved in good quality decision-making. At time, uncertainties about prognosis and lack of knowledge about services and interventions meant carers and the people they cared for felt they did not have the necessary knowledge to take informed decisions.^32,38,54^ Some studies pointed to a need for ongoing flexibility regarding decision making, taking decisions in the moment.^33,47^ Young people and non-spousal carers sometimes reported exclusion from formal decision making.^61,62^ A move into residential care could result in a further change to decision-making roles, with the carer becoming less involved in decision making.^26^

### Maintaining continuity while facing change and disruption

Families, including carers, placed a high value on maintaining a sense of normality in a context where life was far from normal, they were facing unfamiliar and hard-to-predict challenges, and prior understandings of family and relationship norms were disrupted by continual change.^27,33–36,47,48,53^ It was important for family carers to strive to preserve significant personal, couple and family continuities wherever possible^37,38,53^ and to try to establish a routine in order to preserve some sense of control.^63^

However, there were significant challenges for families in their efforts to preserve continuities. Taking on roles and responsibilities that had been the responsibility of the person living with the long-term neurological condition added to workload. Often, spouse/partner carers became solely responsible for all aspects of the household, representing a disruption to established roles and an additional demand on time.^63^ Alternatively, formal carers or other professionals coming into the home could result in a loss of personal or family space.^64^ The growing likelihood of providing complex care meant that carers’ own needs risked becoming incidental and carers often experienced guilt if they expressed or tried to realise their own needs.^31,53,63^ Complex care provided against a backdrop of change and uncertainty reduced the time available to retain important continuities such as hobbies, work or maintaining friendships and family relationships.^63^ Extra effort was involved to engage in activities with the person with long-term neurological conditons.^29,30,62^ Wider family and friendship networks suffered as time, energy and the resources to invest in maintaining those relationships was compromised by the demands of providing care.^26^

Commitment to the relationship and loving the person living with the condition was a key motivation for providing complex and often relentless care.^29,31,37,47^ A study of carers for relatives with Amyotrophic Lateral Sclerosis found that the diagnosis drew some families together but pulled others further apart – the latter occurring more frequently amongst families who avoided emotional content in their communication.^24^ But inevitably, the challenges associated with caring affected relationships. Caring spouses and partners expressed feelings of existential loneliness as they felt unable to share their experiences, worries and thoughts with their spouse/partner, including how the neurological conditions was impacting on each other.^5,26,31,51^ Physical and emotional changes such as pain, reduced mobility, muscle weakness and low mood impacted on the extent to which the person with the condition and their partner could respond to physical closeness and intimacy which, combined with a changing role from partner/spouse to carer, affected sexual and intimate relationships.^48,63^ Changes in a person’s behaviour or mood could have a significant impact on partner relationships and also the wider family and friendships.^26,35,49^

Anticipated loss and perpetual loss was identified as the backdrop for carers of people with long-term neurological conditions,^35,48,63^ creating an intensely challenging situation for them both and their families. Bereavement for some carers was preceded by distressing and difficult end of life experiences^44,56,64^ which carried the risk of a long-lasting effect on the bereaved person. Difficult end of life experiences risked exacerbating feelings such as remorse, blame and guilt as to whether they or others could have done more.^63^ Research has suggested that those spouses/partners who had difficulties accepting that their partner’s condition was terminal were more likely to subsequently have prolonged and complex grief reactions.^48^ Those people who were isolated in their caring role may be more at risk of feeling abandoned or unsupported in bereavement.^10^ Overall, access to bereavement support was patchy and identified as a priority area of need, especially for carers of people with conditions which have tended to be overlooked in the bereavement literature (for example, Parkinson’s Disease).^10,34^

## Discussion

This review addressed the experiences of carers of people with long-term neurological conditions regarding end-of-life. Although papers addressed a range of different issues, the interpreted line of argument highlighted themes of: the future (un)certainties in the progression of life-limiting neurological conditions; an information paradox of both too much and too little information; access to support; carers’ roles in decision making around end of life; and maintaining continuity while facing change and disruption in day-to-day living.

There was a strong sense of ‘uncharted territory’ – long-term neurological conditions were outside the range of expected experience. As a consequence, carers struggled to balance maintaining existing continuities, within the context of an unpredictable trajectory of change and against the emotional backdrop associated with diagnosis of a long-term neurological condition. One of the inevitable limitations of carer research is that it tends to homogenise the needs of carers. This review has highlighted diversity and the variability of information needs. Not all carers wanted the same information at the same time. Carers did not always know what services were available and sometimes only realised what would have been helpful to them retrospectively, suggesting a greater need to engage with carers in order to understand and meet individual needs. Many studies highlighted carers’ frustrations at being expected to navigate services with little signposting. In other cases, support interventions may themselves have represented a significant loss of continuity – for example, accepting that gastrostomy insertion meant that the individual would not eat again, with consequent impact on family life and friends; or the emotional and relationship challenges involved in accepting respite care or domiciliary care.^32,39,53,54,65^

In attempting to make sense of this unfamiliar context, carers at times contrasted neurological conditions to other life-limiting diagnoses, particularly cancer. This occurred both within practical discussions of information and service provision (e.g. that palliative care was frequently perceived as being primarily for cancer care), and within conceptual discussions of disease trajectories.^10,46,59^ Carers appeared to perceive end-of-life with a neurological condition as being worse than other end of life experiences. It is possible that this simply reflects carers’ familiarity with neurological conditions: many carers may not have been in a position to make a true comparison with other end-of-life experiences. Nonetheless, carers perceived that the end-of-life care their loved ones was receiving was worse than it ought to be, sometimes resulting in a sense of guilt or regret.^56^ Some aspects of negative comparison, such as uncertainty over prognosis and timing, are inherent to some of the neurological conditions involved. However, many of the practical points of dissatisfaction, such as poor access to services, difficulty knowing who to talk to and end of life care decisions not being respected, could be addressed by improving palliative pathways and protocols and improving access to bereavement services.

Carers often acted as an information broker, transmitting information between the person being cared for and health and social care practitioners, and this responsibility often increased as the condition deteriorated.^10,21,23,52,53^ At times of crisis, carers were often responsible for attempting to ensure that stated wishes on issues such as resuscitation were respected.^22,42^ The majority of studies included in this review focused on a single carer, typically a partner. However, within the findings, a number of papers highlighted the complexities of managing information and addressing end-of-life care and decision-making within a family network, for example disagreements between relatives over the correct course of action, attempts to shield children from potentially distressing information, and ambiguity over the role of non-spousal carers.^6,23,24,26,37,40,61,62,66^ This suggests a potential need for further research into the experience of different types of carers at end-of-life, as well as the interactions between different individuals within families and other networks providing informal care.

### Strengths and limitations

This was a metaethnographic review that considered a range of qualitative papers on carers’ experiences of end-of-life care with neurological conditions, identifying key, cross-cutting themes within the literature. However, data collection within identified papers took place mainly within the ‘Global North’, predominantly with spousal carers, and studies often used similar approaches to recruiting participants. It is therefore likely that the experiences of some groups of carers – those in the Global South, non-spousal carers and those not in contact with formal support organisations, among others – are underrepresented here. The review consciously omitted dementia, and the search strategy may also have overlooked papers on some less common neurological conditions. These are limitations on our findings.

## Conclusion

The findings of this review suggest that informal carers supporting people with progressive neurological conditions at end of life face particular challenges with regard to uncertainty, variability and discontinuity. Progressive neurological conditions did not align with carer expectations of how end of life ‘should’ be, and end of life services were often perceived to be designed for other conditions, typically cancer. As highlighted by Gofton et al., carers were often dealing with multiple dimensions of uncertainty, some of which were inherent to the condition while others derived from inconsistent access to information and support.^28^ Carers struggled to maintain a sense of continuity in the face of these challenges, and as their loved one’s conditions progressed, were often increasingly expected to take a role in key end-of-life decisions. Although the majority of studies primarily focused on a single carer, typically a partner, neurological conditions often had impacts across a much wider family network, with complexities around the sharing of information.

Most papers that made recommendations for action concluded with a general call for earlier and easier access to information, carer support and palliative care. However, Bentley and O’Connor suggest that, given the specific complexities of neurological conditions, it may be more appropriate to embed palliative approaches in neurological care.^50^ One intervention study that aimed to improve palliative care for people living with neurological conditions and their carers found that in practice, an approach based around having a team to help co-ordinate services and signpost was frustrated by a fundamental lack of other services.^53^ Other studies that focused on specific end-of-life interventions such as advanced care plans and non-invasive ventilation found that lack of awareness by other professionals was sometimes a problem, resulting in issues such as inappropriate attempts to resuscitate.^22,43^ However, another study found that carers welcomed targeted support assessments.^60^

Given the relatively clear narrative regarding existing challenges and frustrations, there is a need for future research focusing on the practical delivery of improved services for carers of people with long-term neurological conditions. There are a number of potential models for doing so, and this is likely to be dependent on the local service context. A key consideration to explore would be the extent to which carers require access to support with specific knowledge of neurological conditions, or whether adaptations can be made to existing carer and palliative services to improve their response to the needs of people living with neurological conditions and their carers.

## References

[1] Feigin VL Abajobir AA Abate KH, et al Global, regional, and national burden of neurological disorders during 1990–2015: a systematic analysis for the Global Burden of Disease Study 2015. Lancet Neurol 2017; 16: 877–897. 28931491 10.1016/S1474-4422(17)30299-5 PMC5641502

[2] Thomas S Davies A Peel C. A mid-term review of the NSF for long-term neurological conditions. Br J Neurosci Nurs 2010; 6: 366–370.

[3] Aubeeluck AV Buchanan H Stupple EJ. ‘All the burden on all the carers’: exploring quality of life with family caregivers of Huntington’s disease patients. Qual Life Res 2012; 21: 1425–1435. 22081218 10.1007/s11136-011-0062-x

[4] National End of Life Care Programme. End of life care in long term neurological conditions: a framework for implementation. London: National Council for Palliative Care/Neurological Alliance, 2010.

[5] Boersma I Jones J Coughlan C, et al Palliative care and Parkinson’s disease: caregiver perspectives. J Palliat Med 2017; 20: 930–938. 28520498 10.1089/jpm.2016.0325 PMC5576067

[6] Ebrahimi H Hasankhani H Namdar H, et al Impacts of chronic illness on families: experiences of Iranian family of patients with Multiple Sclerosis: a qualitative study. J Res Med Dental Sci 2017; 5: 13–18. 10.1155/2017/9243161 PMC5610797 29082042

[7] Fox S Cashell A Kernohan WG, et al Palliative care for Parkinson’s disease: patient and carer’s perspectives explored through qualitative interview. Palliat Med 2017; 31: 634–641. 27683476 10.1177/0269216316669922

[8] Shakespeare J Anderson J. Huntington’s disease–falling through the net. Health Trends 1993; 25: 19–23. 10125696

[9] Draper A Day E Garrood E, et al Patients and carers experience of living with a complex neurological and palliative diagnosis. Mortality 2013; 18: 270–289.

[10] Hasson F Kernohan WG McLaughlin M, et al An exploration into the palliative and end-of-life experiences of carers of people with Parkinson’s disease. Palliat Med 2010; 24: 731–736. 20525749 10.1177/0269216310371414

[11] Skelly R Lindop F Johnson C. Multidisciplinary care of patients with Parkinson’s disease. Prog Neurol Psychiatry 2012; 16: 10–14.

[12] Toze M Sisson K George T, et al Support for people with long-term neurological conditions in rural English communities. Br J Community Nurs 2019; 24: 212–215. 31059297 10.12968/bjcn.2019.24.5.212

[13] Plouvier AOA Olde Hartman TC Verhulst CEM, et al Parkinson’s disease: patient and general practitioner perspectives on the role of primary care. Fam Pract 2016; 34: 227–233. 10.1093/fampra/cmw115 28419289

[14] National Audit Office. Services for people with neurological conditions. London: Department of Health, 2011.

[15] Noblit GW Hare RD. Meta-ethnography: synthesizing qualitative studies. Newbury Park, CA; London: Sage, 1988.

[16] Britten N Campbell R Pope C, et al Using meta ethnography to synthesise qualitative research: a worked example. J Health Serv Res Pol 2002; 7: 209–215. 10.1258/135581902320432732 12425780

[17] Atkins S Lewin S Smith H, et al Conducting a meta-ethnography of qualitative literature: lessons learnt. BMC Med Res Methodol 2008; 8: 21. 18416812 10.1186/1471-2288-8-21 PMC2374791

[18] Greenwood N Mackenzie A. Informal caring for stroke survivors: meta-ethnographic review of qualitative literature. Maturitas 2010; 66: 268–276. 20430542 10.1016/j.maturitas.2010.03.017

[19] Sarmento VP Gysels M Higginson IJ, et al Home palliative care works: but how? A meta-ethnography of the experiences of patients and family caregivers. BMJ Support Palliat Care. Epub ahead of print February 2017. DOI: 10.1136/bmjspcare-2016-001141. 28232515

[20] Larun L Malterud K. Identity and coping experiences in Chronic Fatigue Syndrome: a synthesis of qualitative studies. Patient Educ Couns 2007; 69: 20–28. 17698311 10.1016/j.pec.2007.06.008

[21] O’Brien MR Preston H. Family carer perspectives of acute hospital care following a diagnosis of motor neuron disease: a qualitative secondary analysis. BMJ Support Palliat care 2015; 5: 503–509. 10.1136/bmjspcare-2013-000627 24681558

[22] Preston H Fineberg IC Callagher P, et al The preferred priorities for care document in motor neurone disease: views of bereaved relatives and carers. Palliat Med 2012; 26: 132–138. 21383060 10.1177/0269216311399664

[23] O’Brien MR Whitehead B Jack BA, et al The need for support services for family carers of people with motor neurone disease (MND): views of current and former family caregivers a qualitative study. Disabil Rehabil 2012; 34: 247–256. 22087569 10.3109/09638288.2011.605511

[24] Cipolletta S Amicucci L. The family experience of living with a person with amyotrophic lateral sclerosis: a qualitative study. Int J Psychol 2015; 50: 288–294. 25043818 10.1002/ijop.12085

[25] Lerum SV Solbrække KN Holmøy T, et al Unstable terminality: negotiating the meaning of chronicity and terminality in motor neurone disease. Sociol Health Illn 2015; 37: 81–96. 25601066 10.1111/1467-9566.12182

[26] Bowen C MacLehose A Beaumont J. Advanced multiple sclerosis and the psychosocial impact on families. Psychol Health 2011; 26: 113–127. 20204981 10.1080/08870440903287934

[27] Freer S. Motor neurone disease: insight into experience of family carers. End Life Care J 2010; 4: 54–61.

[28] Gofton T Chum M Schulz V, et al Challenges facing palliative neurology practice: a qualitative analysis. J Neurol Sci 2018; 385: 225–231. 29277430 10.1016/j.jns.2017.12.008

[29] Mutch K. In sickness and in health: experience of caring for a spouse with MS. Br J Nurs 2010; 19: 214–219. 20220670 10.12968/bjon.2010.19.4.46782

[30] Oyebode JR Smith H-J Morrison K. The personal experience of partners of individuals with motor neuron disease. Amyotroph Lateral Scler Frontotemporal Degener 2013; 14: 39–43. 22973854 10.3109/17482968.2012.719236

[31] Ozanne AO Graneheim UH Strang S. Struggling to find meaning in life among spouses of people with ALS. Palliat Support Care 2015; 13: 909–916. 24991842 10.1017/S1478951514000625

[32] Stavroulakis T Baird WO Baxter SK, et al Factors influencing decision-making in relation to timing of gastrostomy insertion in patients with motor neurone disease. BMJ Support Palliat Care 2014; 4: 57–63. 10.1136/bmjspcare-2013-000497 24644772

[33] Anderson NH Gluyas C Mathers S, et al “A monster that lives in our lives”: experiences of caregivers of people with motor neuron disease and identifying avenues for support. BMJ Support Palliat Care 2019; 9: e27. 10.1136/bmjspcare-2015-001057 27125270

[34] Flemming K Turner V Bolsher S, et al The experiences of, and need for, palliative care for people with motor neurone disease and their informal caregivers: a qualitative systematic review. Palliat Med 2020; 34: 708–730. 32286157 10.1177/0269216320908775 PMC7444021

[35] Francis SR Hall EOC Delmar C. Ethical dilemmas experienced by spouses of a partner with brain tumour. Nurs Ethics 2020; 27: 587–597. 31319743 10.1177/0969733019857790

[36] Lu Q Mårtensson J Zhao Y, et al Living on the edge: family caregivers’ experiences of caring for post-stroke family members in China: a qualitative study. Int J Nurs Stud 2019; 94: 1–8. 30928717 10.1016/j.ijnurstu.2019.02.016

[37] Rademeyer M. A stroke of grief and devotion: a hermeneutic enquiry of a family’s lived-experience two years post-stroke. Nurs Prax Aotearoa N Z 2020; 36: 8–19.

[38] Warrier MG Sadasivan A Polavarapu K, et al Lived experience of spouses of persons with motor neuron disease: preliminary findings through interpretative phenomenological analysis. Indian J Palliat Care 2020; 26: 61–65. 10.4103/IJPC.IJPC_123_19 PMC7017690 32132786

[39] Veronese S Gallo G Valle A, et al The palliative care needs of people severely affected by neurodegenerative disorders: a qualitative study. Prog Palliat Care 2015; 23: 331–342.

[40] Murray L Butow PN White K, et al Advance care planning in motor neuron disease: a qualitative study of caregiver perspectives. Palliat Med 2016; 30: 471–478. 26847526 10.1177/0269216315613902

[41] Halpin M. Accounts of suicidality in the Huntington disease community. Omega 2012; 65: 317–334. 23115895 10.2190/OM.65.4.e

[42] Ray RA Brown J Street AF. Dying with motor neurone disease, what can we learn from family caregivers? Health Expect 2014; 17: 466–476. 22512686 10.1111/j.1369-7625.2012.00773.x PMC5060749

[43] Baxter SK Baird WO Thompson S, et al The use of non-invasive ventilation at end of life in patients with motor neurone disease: a qualitative exploration of family carer and health professional experiences. Palliat Med 2013; 27: 516–523. 23462702 10.1177/0269216313478449

[44] Warrier MG Thomas PT Sadasivan A, et al Family caregivers’ experiences with dying and bereavement of individuals with motor neuron disease in India. J Soc Work End Life Palliat Care 2019; 15: 111–125. 31373263 10.1080/15524256.2019.1645081

[45] Veronese S Valle A Chiò A, et al The last months of life of people with amyotrophic lateral sclerosis in mechanical invasive ventilation: a qualitative study. Amyotroph Lateral Scler Frontotemporal Degener 2014; 15: 499–504. 24863495 10.3109/21678421.2014.913637

[46] Penrod J Hupcey JE Baney BL, et al End-of-life caregiving trajectories. Clin Nurs Res 2011; 20: 7–24. 20876553 10.1177/1054773810384852 PMC3756496

[47] Appleton D Robertson N Mitchell L, et al Our disease: a qualitative meta-synthesis of the experiences of spousal/partner caregivers of people with multiple sclerosis. Scand J Caring Sci 2018; 32: 1262–1278. 30144143 10.1111/scs.12601

[48] Aoun SM Connors SL Priddis L, et al Motor neurone disease family carers’ experiences of caring, palliative care and bereavement: an exploratory qualitative study. Palliat Med 2012; 26: 842–850. 21775409 10.1177/0269216311416036

[49] Golla H Mammeas S Galushko M, et al Unmet needs of caregivers of severely affected multiple sclerosis patients: a qualitative study. Palliat Support Care 2015; 13: 1685–1693. 26081132 10.1017/S1478951515000607

[50] Bentley B O’Connor M. The end-of-life experiences of people with motor neuron disease: family carers’ perspectives. J Palliat Med 2016; 19: 857–862. 27135175 10.1089/jpm.2015.0538

[51] Davies F Edwards A Brain Ka, et al ‘You are just left to get on with it’: qualitative study of patient and carer experiences of the transition to secondary progressive multiple sclerosis. BMJ Open 2015; 5: e007674. 10.1136/bmjopen-2015-007674 PMC4513516 26201723

[52] Winther D Kirkegaard Lorenzen C Dreyer P. Everyday life experiences of close relatives of people with amyotrophic lateral sclerosis receiving home mechanical ventilation—a qualitative study. J Clin Nurs 2020; 29: 2306–2316. 32159905 10.1111/jocn.15239

[53] Lerum SV Solbrække KN Frich JC. Family caregivers’ accounts of caring for a family member with motor neurone disease in Norway: a qualitative study. BMC Palliat Care 2016; 15: 22. 26911713 10.1186/s12904-016-0097-4 PMC4765180

[54] Payne S Burton C Addington-Hall J, et al End-of-life issues in acute stroke care: a qualitative study of the experiences and preferences of patients and families. Palliat Med 2010; 24: 146–153. 19926644 10.1177/0269216309350252

[55] Wallengren C Segesten K Friberg F. Relatives’ information needs and the characteristics of their search for information–in the words of relatives of stroke survivors. J Clin Nurs 2010; 19: 2888–2896. 20846233 10.1111/j.1365-2702.2010.03259.x

[56] Whitehead B O’Brien MR Jack BA, et al Experiences of dying, death and bereavement in motor neurone disease: a qualitative study. Palliat Med 2012; 26: 368–378. 21712334 10.1177/0269216311410900

[57] O’connor M Aoun SM Breen LJ. Australian family carer responses when a loved one receives a diagnosis of Motor Neurone Disease—“our life has changed forever”. Health Soc Care Community 2018; 26: e415–e421. 10.1111/hsc.12541 29359485

[58] Mc Veigh C Donaghy C Mc Laughlin B, et al Palliative care for patients with motor neurone disease and their bereaved carers: a qualitative study. BMC Palliat Care 2019; 18: 1–8. 31027498 10.1186/s12904-019-0423-8 PMC6486679

[59] McLaughlin D Hasson F Kernohan WG, et al Living and coping with Parkinson’s disease: perceptions of informal carers. Palliat Med 2011; 25: 177–182. 20952448 10.1177/0269216310385604

[60] Aoun SM. Bereavement support: from the poor cousin of palliative care to a core asset of compassionate communities. Prog Palliat Care 2020; 28: 107–114.

[61] Kavanaugh MS Noh H Zhang L. Caregiving youth knowledge and perceptions of parental end-of-life wishes in Huntington’s disease. J Soc Work End Life Palliat Care 2016; 12: 348–365. 27938026 10.1080/15524256.2016.1252828

[62] McCurry MK. An exploratory study of decision making by informal caregivers of individuals with multiple sclerosis. J Neurosci Nurs 2013; 45: 52–60. 23291872 10.1097/JNN.0b013e318275b252

[63] Mantell A. Under a cloud: carers’ experiences of Huntington’s disease. Soc Care Neurodisability 2010; 1: 33–42.

[64] Harris DA Jack K Wibberley C. Making her end of life her own: further reflections on supporting a loved one with motor neurone disease. Int J Palliat Nurs 2019; 25: 284–292. 31242093 10.12968/ijpn.2019.25.6.284

[65] Weisser FB Bristowe K Jackson D. Experiences of burden, needs, rewards and resilience in family caregivers of people living with motor neurone disease/amyotrophic lateral sclerosis: a secondary thematic analysis of qualitative interviews. Palliat Med 2015; 29: 737–745. 25762578 10.1177/0269216315575851

[66] Giovannetti AM Borreani C Bianchi E, et al Participant perspectives of a home-based palliative approach for people with severe multiple sclerosis: a qualitative study. PLoS One 2018; 13: e0200532. 10.1371/journal.pone.0200532 PMC6042757 30001423

[67] Aoun SM Deas K Kristjanson LJ, et al Identifying and addressing the support needs of family caregivers of people with motor neurone disease using the Carer Support Needs Assessment Tool. Palliat Support Care 2017 2; 15: 32–43. DOI: 10.1017/S1478951516000341. 27173737

